# Morphologic and Functional Effects of Gamma Secretase Inhibition on Splenic Marginal Zone B Cells

**DOI:** 10.1155/2012/289412

**Published:** 2012-12-17

**Authors:** Maria Cristina de Vera Mudry, Franziska Regenass-Lechner, Laurence Ozmen, Bernd Altmann, Matthias Festag, Thomas Singer, Lutz Müller, Helmut Jacobsen, Alexander Flohr

**Affiliations:** ^1^Non-Clinical Safety, Pharma Research and Early Development, F. Hoffmann-La Roche Ltd., Grenzacherstrasse 124, 4070 Basel, Switzerland; ^2^Molecular Neuroscience, Pharma Research and Early Development, F. Hoffmann-La Roche Ltd., Grenzacherstrasse 124, 4070 Basel, Switzerland; ^3^Medicinal Chemistry, Pharma Research and Early Development, F. Hoffmann-La Roche Ltd., Grenzacherstrasse 124, 4070 Basel, Switzerland

## Abstract

The **γ**-secretase complex is a promising target in Alzheimer's disease because of its role in the amyloidogenic processing of **β**-amyloid precursor protein. This enzyme also catalyzes the cleavage of Notch receptor, resulting in the nuclear translocation of intracellular Notch where it modulates gene transcription. Notch signaling is essential in cell fate decisions during embryogenesis, neuronal differentiation, hematopoiesis, and development of T and B cells, including splenic marginal zone (MZ) B cells. This B cell compartment participates in the early phases of the immune response to blood-borne bacteria and viruses. Chronic treatment with the oral **γ**-secretase inhibitor RO4929097 resulted in dose-dependent decreased cellularity (atrophy) of the MZ of rats and mice. Significant decreases in relative MZ B-cell numbers of RO4929097-treated animals were confirmed by flow cytometry. Numbers of MZ B cells reverted to normal after a sufficient RO4929097-free recovery period. Functional characterization of the immune response in relation to RO4929097-related MZ B cell decrease was assessed in mice vaccinated with inactivated vesicular stomatitis virus (VSV). Compared with the immunosuppressant cyclosporin A, RO4929097 caused only mild and reversible delayed early neutralizing IgM and IgG responses to VSV. Thus, the functional consequence of MZ B cell decrease on host defense is comparatively mild.

## 1. Introduction

The *γ*-secretase complex is a key enzyme in the amyloidogenic processing of *β*-amyloid precursor protein (APP) and is a promising drug target for treatment or prevention of Alzheimer's disease [1]. In the first step, the extracellular domain of APP is cleaved off by the “sheddase” activity of either an ADAM protease or *β*-secretase. The remaining membrane-bound C-terminal fragment is then cleaved by *γ*-secretase within its transmembrane domain. The cleavage releases a cytoplasmic domain into the cell interior, but produces also the *β*-amyloid peptides which are released into the extracellular space where they can form toxic aggregates and amyloid plaques. The latter presumably initiate a pathogenic cascade which finally leads to the clinical symptoms of Alzheimer's disease, that is, severe dementia.

A number of additional *γ*-secretase substrates have been identified and among these are the Notch receptors, CD44 and HER4 [2, 3]. These are all type-1 membrane proteins and their processing follows the same pattern as described for APP. In the case of Notch, the shedding of the extracellular domain is initiated by binding of specific ligands like Delta-like and Jagged. Once released by *γ*-secretase from its membrane attachment, the cytoplasmic domain of Notch (ICN, intracellular cellular Notch) translocates to the nucleus where it serves as a transcriptional regulator. Notch signaling is important for cell fate decisions and homeostasis of pluripotent stem cells in the embryo and adult organism [4] and is upregulated in neoplastic cells [5]. Targeted inactivation of the Notch pathway can affect processes such as the renewal of epidermis and gut epithelia and specific populations of lymphocytes, as well as tumors. For this reason, pharmacological inhibition of *γ*-secretase, for example, as a potential treatment in Alzheimer's disease or neoplasia, can have adverse effects on important physiological processes because of its effects on Notch signaling [6–9].

One group of B lymphocytes that depends on active Notch signaling is the marginal zone B cell [10]. In the current report, we investigated the effects of a selective and potent *γ*-secretase inhibitor (GSI), RO4929097 [11], on the formation and function of MZ B cells in the rat and mouse *in vivo*. Specifically, the effect of RO4929097 on (1) the number of MZ B cells in the spleen in rats after 13 weeks of oral administration followed by a treatment-free recovery period of 6 weeks, and on (2) the neutralizing antibody formation after challenge with inactivated vesicular stomatitis virus (VSV) in mice after 4 weeks of oral administration was assessed.

## 2. Materials and Methods

### 2.1. Compound

The test compound RO4929097 [2,2-dimethyl-N-((S)-6-oxo-6,7-dihydro-5H-dibenzo[b,d]azepin-7-yl)-N-(2,2,3,3,3-pentafluoro-propyl)-malonamide] was synthesized according to the procedure described in patent application (WO2005/023772).

### 2.2. Animals

Male and female Wistar (HanRCCWIST) SPF rats, 12 weeks of age, and female C57Bl/6JIbm SPF mice, 8–10 weeks of age, (RCC, Füllinsdorf, Switzerland), were used. Animals were provided with pelleted maintenance rodent diet Provimi Kliba 3433 and tap water *ad libitum. *Rats were kept 2 per cage, in Makrolon type III cages, while mice were kept individually in Makrolon type II cages, with autoclaved sawdust bedding. Air-conditioned animal rooms were maintained at 22°C ± 2°C and 40%–80% relative humidity, under periodic bacteriological control. A 12-h light/dark cycle was used and background music was coordinated with light hours. Animals were handled with humane care according to the guidelines of the Institutional Animal Care and Use Committee. The F. Hoffmann-La Roche Pharma Research Basel test facility is certified by the Swiss GLP monitoring authorities to be in compliance with the Swiss Ordinance relating to Good Laboratory Practice (GLP) and is fully accredited by the Association for Assessment and Accreditation of Laboratory Animal Care International.

### 2.3. Treatments

Rats were administered RO4929097, a potent and selective *γ*-secretase inhibitor, in the vehicle (Capmul/MCT/Tween 80 solution) at doses of 0, 0.5, 1.5, and 3 mg/kg/day, orally (gavage), once daily for 13 weeks, at a dose volume of 1 mL/kg body weight. Rats were kept for an additional 6 weeks of treatment-free recovery period.

For the VSV vaccination study, female C57Bl/6JIbm mice were administered RO4929097 in 3% Avicel and 0.2% Tween 80 at doses of 0, 3, 10, and 30 mg/kg/day orally (gavage), once daily for 4 weeks. Half of the mice received in addition 60 mg/kg/day cyclosporin A (Sandimmun Neoral, Novartis Pharma) orally from day 5. Ultraviolet (UV) light-inactivated VSV Indiana was kindly provided by Dr. D. Pinschewer (Institute of Experimental Immunology, University Hospital Zürich, Switzerland). All mice were immunized with 4 × 10^6^ PFU equivalents UV-inactivated VSV intravenously on day 9. Serum samples were obtained at different time points to monitor VSV-specific antibody production. Due to technical difficulties during the dosing procedure, some animals did not survive the duration of the study. This resulted in the analysis of 5 mice in the vehicle control group, and 8 mice each in the treated groups.

### 2.4. Terminal Procedures

Animals were sacrificed with CO_2_ and exsanguinated. One-third of the spleen of all animals was taken and placed in 5 mL cold sterile PBS, stored on ice until processing for marginal zone B-cell enumeration. The remainder of the spleen was fixed for at most 24 h in 10% buffered formalin, embedded in Paraplast, sectioned at a nominal thickness of approximately 2–4 *μ*m, and stained for H.E. for routine microscopic examination.

### 2.5. Flow Cytometric Analysis of Marginal Zone B Cells

Spleen cell suspensions were prepared by passing through a 70 *μ*m cell strainer in cold DMEM (Dulbecco's Minimal Essential Medium). Red blood cells were lysed and washed cells were incubated with a mixture of the following antibodies and acquired in a FACSCanto or FACSCalibur instrument (Becton Dickinson). Analysis was performed using CellQuest software (Becton Dickinson) and results were expressed as percentages of MZ B cells within the leukocyte gate.

**Mouse**

FITC-conjugated anti-mouse CD21/CD35 (BD Pharmingen), PE-conjugated anti-mouse CD23 (BD Pharmingen), Cy5.5-conjugated anti-mouse B220 (eBioscience), APC-conjugated anti-mouse IgM (BD Pharmingen) were used. MZ B cells were characterized as being IgM^+^/B220^+^/CD21^high^/CD23^low^.

**Rat**

FITC-conjugated anti-rat HIS57 (BD Pharmingen), PE-conjugated anti-rat IgM (BD Pharmingen), PerCP-conjugated anti-rat Thy1.1 (CD90) (BD Pharmingen) were used. MZ B cells were characterized as IgM^high^/CD90^low/negative^/HIS57^+^.

### 2.6. VSV Neutralization Assay

After intravenous immunization with 2 × 10^6^ PFU (plaque-forming units) VSV-IND, serum was collected from mice at specified times (days 2, 4, 7, 14, and 20 after immunization). In the morning before dosing with RO4929097, approximately 200 *μ*L blood was drawn retroorbitally from mice under light isoflurane anesthesia, centrifuged for 1 h, and the serum frozen at −20°C. Thawed serum was prediluted 40-fold in DMEM containing 5% FCS and heat-inactivated for 30 min at 56 °C. Serial twofold dilutions were mixed with equal volumes of VSV (500 PFU mL^−1^) and incubated for 90 min at 37 °C in an atmosphere with 5% CO_2_. The serum-virus mixture was transferred onto Vero cell monolayers in 96-well plates and incubated for 1 h at 37 °C. The monolayers were then overlaid with DMEM containing 1% methylcellulose and incubated for 24 h at 37 °C after which the monolayers were fixed and stained with 0.5% crystal violet. The highest dilution of serum that reduced the number of plaques by 50% was taken as the titer. To determine IgG titers, undiluted serum was pretreated with an equal volume of 0.1 mM *β*-2-mercaptoethanol in saline before heat inactivation and plating onto Vero cells. This treatment has been shown to eliminate only IgM but not IgG from serum [12]. Unreduced samples were taken as IgM titers only, if the corresponding reduced samples had at least a fourfold lower titer, that is, when the IgG present in the unreduced sample could be neglected.

### 2.7. Statistics

For significance testing of dose-related effects on cellularity of MZ B cells in spleen and VSV neutralizing antibody titers in serum, Student's *t*-test was used. Individual time points (study days) were analyzed separately.

For significance testing of dose-related effects on white blood cell, neutrophil, and lymphocyte numbers in peripheral blood, Jonckheere and the Mann-Whitney *U* tests were used.

## 3. Results

### 3.1. 13-Week RO4929097 Treatment in Rats Followed by a 6-Week Treatment-Free Recovery Period

Oral administration of RO4929097 for 13 weeks was generally well tolerated and there were no treatment-related deaths during the course of the study. In the peripheral blood, moderate reductions in lymphocytes and increases in neutrophils from ≥1.5 mg/kg/day in both male and female rats persisted until the end of the 6-week treatment-free recovery period (Tables 1(a) and 1(b)).

**Figure 1:**
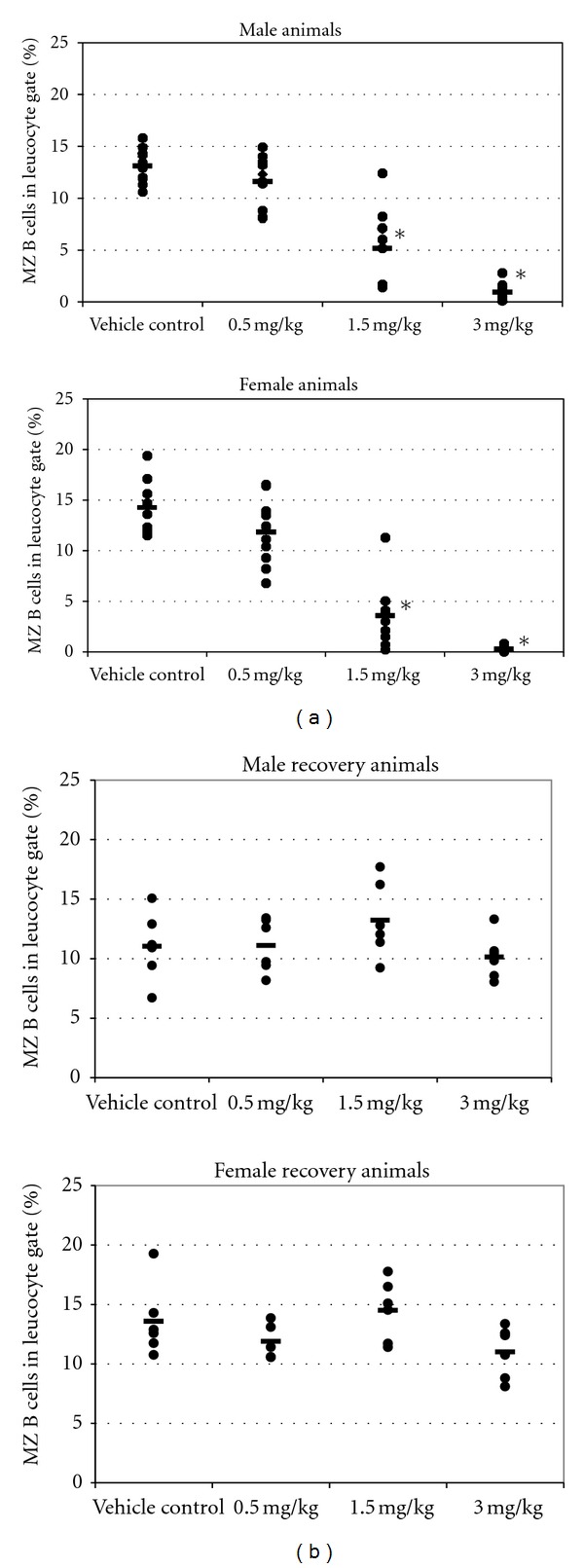
(a) Marginal zone B cells in male and female rats dosed for 13 weeks with RO4929097. Spleen cell suspensions were analyzed by flow cytometry (FACS) using fluorescently labeled antibodies. For each rat the percentage of MZ B cells (= IgM^high^/CD90^low/neg^/HIS57^+^) in the leukocyte gate is shown. Dots represent individual rats. - = average per group. ∗ = statistically significant difference to vehicle control (*P* < 0.05). *n* = 10 rats/sex/group. (b) Splenic MZ B cells in male and female rats dosed for 13 weeks with RO4929097 followed by a recovery phase of 6 weeks. Dots represent individual rats. **- **= average per group. *n* = 6 rats/sex/group.

Flow cytometry analysis revealed a dose-dependent reduction in the percentage of MZ B cells in the spleen of both male and female rats following oral RO4929097 administration for 13 weeks. Compared with the control, this reduction was statistically significant and greater than 60% reduction at ≥1.5 mg/kg/day (Figure 1(a)). After the recovery period, the percentages of marginal zone B cells of RO4929097-treated rats had normalized and were comparable with those of control animals (Figure 1(b)).

**Figure 2:**
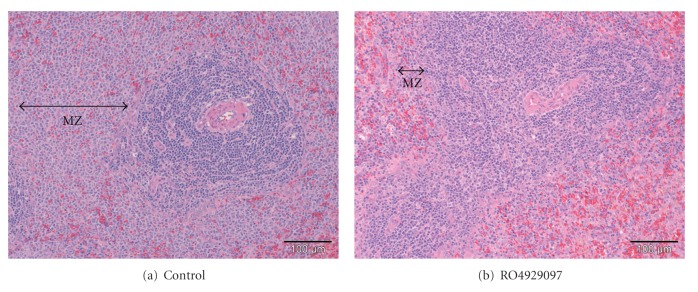
Atrophy of the marginal zone (MZ) of the spleen of rats administered 3.0 mg/kg/day RO4929097 for 13 weeks. (hematoxylin-eosin stain).

Histopathologic changes were recorded in the spleen of nearly all male and female rats that received ≥1.5 mg/kg/day RO4929097 (Figure 2). These included minimal-to-marked decreased cellularity and infiltration of neutrophils of the marginal zone and periarteriolar lymphoid sheath areas. Individual animals showed minimally increased apoptosis in the marginal zone. In addition, slight-to-moderate stromal change, characterized by a focal, somewhat circumscribed area of occasional pale staining lymphocytes, dendritic cells, eosinophilic hyaline-like deposits, early fibroplasia, necrotic cell debris, and polymorphonuclear leukocyte infiltration, was noted in occasional animals. There were no RO4929097-related findings in the spleen after the 6-week treatment-free recovery period.

### 3.2. VSV Vaccination Study in Mice

Mice were treated daily with RO4929097 and vaccinated with inactivated VSV in order to characterize the immune response to VSV in relation to a dose-dependent decrease in MZ B cellularity. The immunosuppressant cyclosporin A was used as a positive control. Cyclosporin A is known to suppress the switch from a T-independent IgM to a T-dependent IgG response to VSV in this model [13].

**Figure 3:**
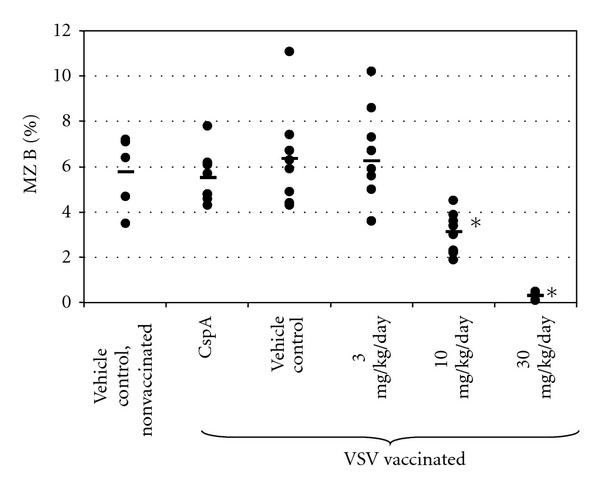
Marginal zone B cells in mice dosed with RO4929097 for 27 days. For each mouse the percentage of MZ B cells (= IgM^+^/B220^+^/CD21^high^/CD23^low^) in the leukocyte gate is shown. Dots represent individual mice. - = average per group. ∗ = statistically significant difference to vehicle control (*P* < 0.01). CspA = cyclosporin A. *n* = 5–8 mice/sex/group.

Flow cytometry analysis of spleen cells at the end of the 4-week treatment period showed a dose-dependent reduction of relative MZ B cell counts (Figure 3). There was no significant reduction at 3 mg/kg/day, a 51% reduction at 10 mg/kg/day, and a 95% reduction at 30 mg/kg/day RO4929097. Vehicle or cyclosporin A treatment had no effect on MZ B cellularity.

**Figure 4:**
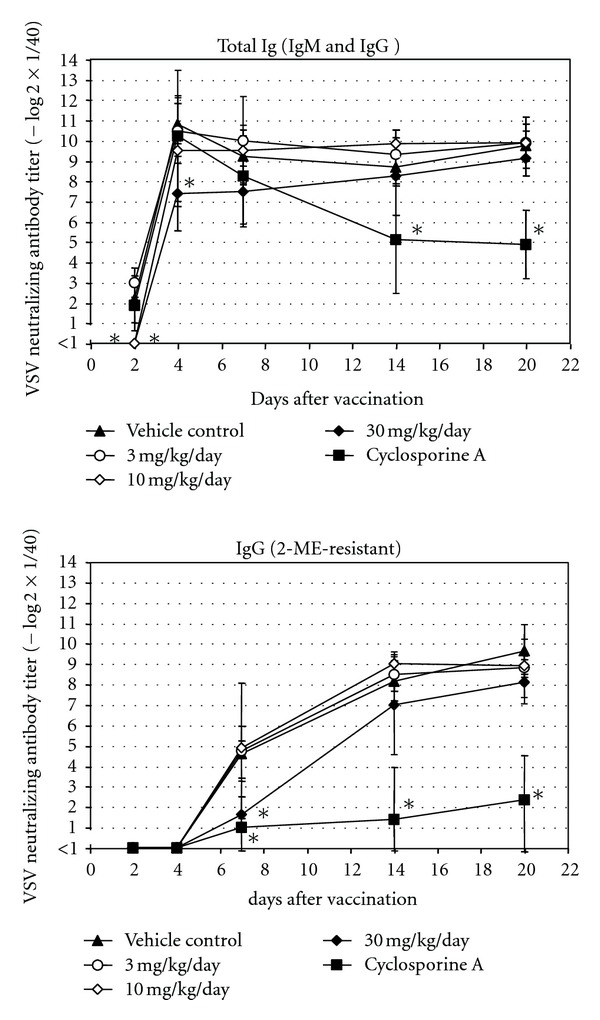
Neutralizing VSV antibodies in mice treated with RO4929097 for 27 days. Neutralizing total Ig (= IgM + IgG) and IgG antibody titers in serum are shown. For each group, average and standard deviation at different time points are shown. ∗ = statistically significant difference to vehicle control (*P* < 0.01) at given time-point. Detection limit of assay was 1 (= 1/40 dilution of serum). Non-vaccinated mice had no detectable VSV neutralizing antibodies in their serum (<1) (not shown). *n* = 4–8 mice/sex/group.

Dosing with ≥10 mg/kg/day RO4929097 resulted in a delayed appearance of early neutralizing antibodies (presumably of IgM class) in response to VSV, that is, titers were below detection limit at 2 days after vaccination (Figure 4). Notably at 10 mg/kg with an average 51% depletion of MZ B cells, the antibody response to VSV was only affected at the earliest time point on day 2, after which no difference to untreated animals in the immune response was noted. At 4 days after vaccination, antibody titers were detectable in all groups, but these were still reduced in animals dosed with 30 mg/kg/day in comparison to all other groups. In addition, treatment with 30 mg/kg/day RO4929097 resulted in a reduction of neutralizing immunoglobulin titers of the IgG class at 7 days after vaccination (average 7.8-fold reduction), but titers recovered at later time points.

## 4. Discussion and Conclusion

Previous preclinical toxicity studies with RO4929097 in rats, mice, and dogs have shown that significant effects were attributable to Notch impairment [11]. Similar findings were observed following oral administration of RO4929097 to male and female Wistar rats for 13 weeks. Flow cytometry analysis showed a dose-dependent reduction in relative numbers of splenic MZ B cells that correlated with histopathologic atrophy of the MZ. Marginal zone B cell depletion was completely reversible after a 6-week recovery period. Similarly, a dose-dependent reduction in relative numbers of splenic MZ B cells was seen in female C57Bl/6JIbm mice administered RO4929097 for 4 weeks. Total early neutralizing antibodies (presumably IgM) to inactivated VSV were also reduced, and this was considered to reflect the pharmacological effect of *γ*-secretase inhibitor RO4929097 on MZ B cells. At the high dose of 30 mg/kg/day RO4929097, neutralizing IgG titers were also reduced at 7 days after vaccination, but recovered at later time points. Class switching from IgM to IgG requires T-cell help, and it is conceivable that high dose RO4929097 not only affected MZ B-cell cellularity but also T-cell function since Notch is also expressed in T cells. Alternatively, a severely reduced MZ B-cell compartment resulted in fewer B cells switching from IgM to IgG. The effect of RO4929097 on IgG antibody formation in these mice was considered relatively mild and a threshold (i.e., 3 mg/kg/day) could be defined where no effects on MZ B-cell counts and neutralizing antibody responses were seen. Originally designed for the treatment of Alzheimer's disease, the efficacious dose of RO4929097 on A*β* reduction was found to be 3 mg/kg in the brain of A*β* transgenic mice, and 10 mg/kg in cerebrospinal fluid of naïve rats. The ED_50_ for mouse MZ B-cell depletion was 10 mg/kg, while for the rat it was lower at 1 mg/kg, but differences in treatment duration should be taken into account. As expected, cyclosporin A showed a much more pronounced effect on neutralizing IgG titers in mice than RO4929097, which was likely due to the known immunosuppressive effect of cyclosporin A on T helper cells, but not on MZ B cells [13].

Much remains unclear regarding development of marginal zone B cells [14]. The immunological consequences of Notch inhibition *in vivo* are not well characterized, and especially little is known about the host response to pathogens under Notch blockade secondary to GSIs. Increased susceptibility to blood-borne bacterial infection has been demonstrated in RBP-J deficient mice [15]. MZ B cells participate in the early phases of the immune response to blood-borne bacteria and viruses. The immune defense of mice to VSV is strongly dependent on the early formation of neutralizing antibodies which in part originate from MZ B cells [15–17]. Therefore the VSV infection or vaccination model can be used to assess the functionality of MZ B cells (and the total B-cell compartment) *in vivo*.

Although RO4929097 was originally designed for the treatment of Alzheimer's disease, the compound is currently under evaluation in clinical studies for oncology. Phase I and other ongoing studies of RO4929097 show that it is generally well tolerated [18–20]. Dose-limiting toxicities arising from administration of *γ*-secretase inhibitors have been reported as mainly due to effects of Notch inhibition on gut epithelium, rather than on marginal zone B cells, correlating well with preclinical safety studies [6–9]. However, Phase III trials with the *γ*-secretase inhibitor semagacestat were halted partly because of increased risk of skin cancer that might be attributable to inhibition of Notch1 [21].

Pharmacodynamic biomarkers of Notch inhibition utilizing microarray gene expression of plucked hair follicles have been described in rats [11] and humans [22]. While the hair follicle is a more readily accessible tissue for a biomarker assay, it cannot be used to assess the functional reserve of the marginal zone. It will be interesting to explore further the transcriptional effects of GSIs on marginal zone B cells in the rodent spleen, and their counterpart in humans in the peripheral blood.

## Figures and Tables

**Table 1a:** (a) After 13 weeks of treatment

		Males			Females	
Dose (mg/kg/day)	WBC	Neutrophils	Lymphocytes	WBC	Neutrophils	Lymphocytes
	(10^9^/L)	(10^9^/L)	(G/L)	(10^9^/L)	(10^9^/L)	(10^9^/L)
0						
*N *	10	10	10	10	10	10
Mean	5.95	1.23	4.26	3.77	0.55	2.94
SD	1.23	0.42	0.92	0.60	0.20	0.47
0.5						
*N *	10	10	10	10	10	10
Mean	5.65	1.20	4.05	3.68	0.67^#^	2.76
SD	1.08	0.39	0.75	0.92	0.12	0.86
1.5						
*N *	10	10	10	9	9	9
Mean	6.31	1.78*	4.01	3.10	1.11**	1.70**
SD	1.39	0.54	1.28	0.85	0.54	0.78
3						
*N *	10	10	10	10	10	10
Mean	6.37	2.95**	2.70**	2.87	1.10	1.51
SD	1.58	1.29	1.17	0.47	0.24	0.47

Jonckheere test: **P ≤ 1%; *1% < *P* ≤ 5%.

*U* test: *P *≤ 1%; ^#^1% < *P* ≤ 5%.

*N*: number of animals.

SD: standard deviation.

**Table 1b:** (b) After 6 weeks of recovery

		Males			Females	
Dose (mg/kg/day)	WBC	Neutrophils	Lymphocytes	WBC	Neutrophils	Lymphocytes
	(10^9^/L)	(10^9^/L)	(10^9^/L)	(10^9^/L)	(10^9^/L)	(10^9^/L)
0						
*N *	10	10	10	10	10	10
Mean	7.56	1.02	6.19	5.28	0.68	4.36
SD	1.17	0.27	1.18	0.84	0.18	0.76
0.5						
*N *	10	10	10	10	10	10
Mean	7.28	1.07	5.85	5.06	0.68	4.14
SD	1.37	0.25	1.16	1.00	0.17	0.90
1.5						
*N *	10	10	10	10	10	10
Mean	8.40	1.70**	6.25	4.01**	1.28**	2.45**
SD	1.34	0.50	1.25	1.03	0.72	0.81
3						
*N *	10	10	10	10	10	10
Mean	9.13*	3.45**	4.99	3.85**	1.48**	2.08**
SD	2.32	2.10	1.73	0.78	0.54	0.59

Jonckheere test: **P ≤ 1%; *1% < *P* ≤ 5%.

*N*: number of animals.

SD: standard deviation.

## Abbreviations

APP: *β*-amyloid precursor protein
MZ: Marginal zone
VSV: Vesicular stomatitis virus
GSI: *γ*-secretase inhibitor
FACS: Fluorescence activated cell sorting.
